# Targeting Bruton’s tyrosine kinase in vitreoretinal lymphoma: an open-label, prospective, single-center, phase 2 study

**DOI:** 10.1186/s40164-022-00354-2

**Published:** 2022-11-08

**Authors:** Wenxue Guan, Liang Wang, Xiaoyan Peng

**Affiliations:** 1 Department of Ophthalmology, Beijing Tongren Hospital, Capital Medical University, Beijing, 100005 China; 2 Department of Hematology, Beijing Tongren Hospital, Capital Medical University, Beijing, 100005 China

**Keywords:** Vitreoretinal lymphoma, Bruton’s tyrosine kinase, Treatment, Safety

## Abstract

Vitreoretinal lymphoma (VRL) is strongly linked to central nervous system (CNS) progression with no standard treatment approaches. Commonly used strategies include repeated intraocular injections of low-dose methotrexate or local radiotherapy, with great inconvenience, long-term side effects, and high risk of CNS relapse. In this study, we evaluated the efficacy and safety of bruton’s tyrosine kinase inhibitors (BTKi) in the treatment of VRL. This prospective single-center study enrolled patients with relapsed or newly diagnosed VRL between October 2020 and April 2022. Patients received BTKi monotherapy until disease progression or unacceptable toxicity. The primary endpoint was the disease control (DC) rate after one month of treatment; secondary endpoints include toxicity, overall survival (OS), and progression-free survival (PFS). Ten consecutive patients with VRL were enrolled into this study. After 1-month treatment, 9 patients (90%) achieved a DC, with 7 patients (70%) achieving a complete response (CR). With a median follow-up of 8.3 (2.5–21.4) months, 4 patients were confirmed to have disease progression, with a PFS of 1.2, 7.5, 9.1, and 11.6 months, respectively. The remaining 6 patients have durable control of disease and were still on treatment at time of the analysis. BTKi were well-tolerated and no patients discontinued the drug because of adverse events. In conclusion, targeting BTK in VRL is viable, and our findings could pave the way for a paradigm change in VRL therapy choices. Further large-scale studies, however, are required to give stronger evidence about the efficacy and safety.

**Supplementary Information:**

The online version contains supplementary material available at 10.1186/s40164-022-00354-2.

To the editor,

Vitreoretinal lymphoma (VRL) is a rare primary central nervous system lymphoma (PCNSL) that affects vitreous and/or retina, 90% of which will eventually progress to CNS involvement, and no optimal treatments have been defined yet [1–3]. Intraocular injection of methotrexate is the most commonly used strategies, with great inconvenience, long-term side effects, and high risk of CNS relapse [4, 5]. Bruton’s tyrosine kinase (BTK) has been validated as a therapeutic target for a variety of B-cell malignancies [6–9]. Ibrutinib, a first-in-class BTK inhibitor (BTKi), was shown to have a 100% disease control rate in 14 isolated VRL patients after two months of treatment [10]. We also reported promising results using Zanubrutinib in PCNSL patients with isolated VRL relapse [11]. Herein, the results of a prospective phase 2 study evaluating the efficacy and safety of BTKi in patients with VRL (ChiCTR2000037921) were reported.

This was an open-label, prospective, phase 2 study approved by IRB of Beijing Tongren Hospital. All individuals had confirmed diagnosis of VRL based on vitreous and/or brain biopsy, and were treated with orally BTKi monotherapy (ibrutinib 560 mg once daily, zanubrutinib 160 mg twice daily or orelabrutinib 150 mg daily), until disease progression or unacceptable toxicity.

Between October 2020 and April 2022, ten patients with VRL were enrolled. Three patients had bilateral VRL and seven patients had unilateral ocular involvement. Six patients were newly diagnosed with VRL, and four previously treated with intravitreal methotrexate had disease relapse in the eye (patients #1, #2, and #10) or combined with the CNS (patient #4). The time to relapse after previous therapies was a median of 4 (1.8–11.8) months. Patients’ characteristics are provided in Additional file 1: Table S1 and diagnostic test results showed in Additional file 2: Table S2.

After 1-month BTKi treatment, 9 out of 10 patients (90%) achieved disease control (DC), including complete remission in 7 patients (70%) with symptoms resolved, vitreous cell disappearance, regression of retinal infiltrates, and interleukin (IL)-10 level normalization in the aqueous humor (AH), and partial remission in 2 cases (20%) with a massive decrease of cell infiltration within the vitreous and retina. Table 1 and Fig. 1 show detailed efficacy data. The mean best-corrected visual acuity (BCVA) of the 13 eyes improved significantly from 0.6 ± 0.4 log MAR (Snellen equivalent 20/80) to 0.4 ± 0.5 (20/50) at one month of follow-up and to 0.4 ± 0.4 (20/50) at last visit (Fig. 1A). The AH IL-10 levels increased in 13 of the 20 examined eyes at the time of diagnosis. After one-month BTKi treatment, all but one patient's IL-10 levels were below the detection limit (5 pg/ml) (Fig. 1B). At a median follow-up of 8.3 (2.5–21.4) months, all patients were alive at the time of this report except for one who had both CNS and ocular relapses prior to BTKi treatment, with an overall survival rate of 90% (Fig. 1C). Four patients were confirmed to have disease progression, with a progression-free survival (PFS) of 1.2, 7.5, 9.1, and 11.6 months, respectively (Fig. 1D). Two patients (#1 and #5) experienced ocular relapses and CNS progression, respectively, after 9.1 months and 11.6 months of continuous BTKi dosing. The remaining 6 patients had durable control of diseases and were still on treatment at time of the analysis (Fig. 1E). BTKi were well-tolerated, with grade 1 ecchymosis in 3 patients and grade 2 arthralgia in 1 patient. No patients discontinued the drug because of adverse events (Additional file 3).

**Table 1:** Treatments and survival characteristics of VRL patients

Patient	BTKi treatment (months)	Initial response (1 month)	Complications	Ocular relapse after initial remission (months)	CNS progression	PFS^#^ (months)	OS* (months)	Survival status	Follow-up ^ꝉ^ (months)
#1	Z (6) O (3)	CR	No	Bilateral eyes (9.1)	No	9.1	21 +	Alive	21
#2	Z (4)	CR	Ecchymosis (grade 1)	Bilateral eyes (7.5)	10 months after BTKi discontinuation	7.5	20 +	Alive	20
#3	O (18)	CR	Ecchymosis (grade 1)	No	No	18 +	18 +	Alive	18
#4	Z (1)	PD	No	No	1.2 months since BTKi treatment	1.2	2	Dead due to CNS progression	2
#5	O (6) Z (6)	PR	Arthralgia (grade2) Ecchymosis (grade 1)	No	11.6 months since BTKi treatment	11.6	12 +	Alive	12
#6	O (9)	CR	No	No	No	9 +	9 +	Alive	9
#7	O (7)	CR	No	No	No	7 +	7 +	Alive	7
#8	I (6)	CR	No	No	No	6 +	6 +	Alive	6
#9	O (3)	PR	No	No	No	3 +	3 +	Alive	3
#10	O (3)	CR	No	No	No	3 +	3 +	Alive	3

BTKi, Bruton tyrosine kinase inhibitors; CNS, central nervous system; CR, complete remission; PD, progressive disease; PFS, progression-free survival; PR, partial remission; I, ibrutinib; O, orelabrutinib; OS, overall survival; Z, zanubrutinib

^#^ PFS was calculated as the period from the onset of BTK inhibitors to lymphoma relapse, death, or the final follow-up

^*^OS was computed from the date of BTKi initiation to the date of the last follow-up or death

^ꝉ^ Follow-up was calculated from the onset of BTK inhibitors until death or the last follow-up

**Fig. 1:**
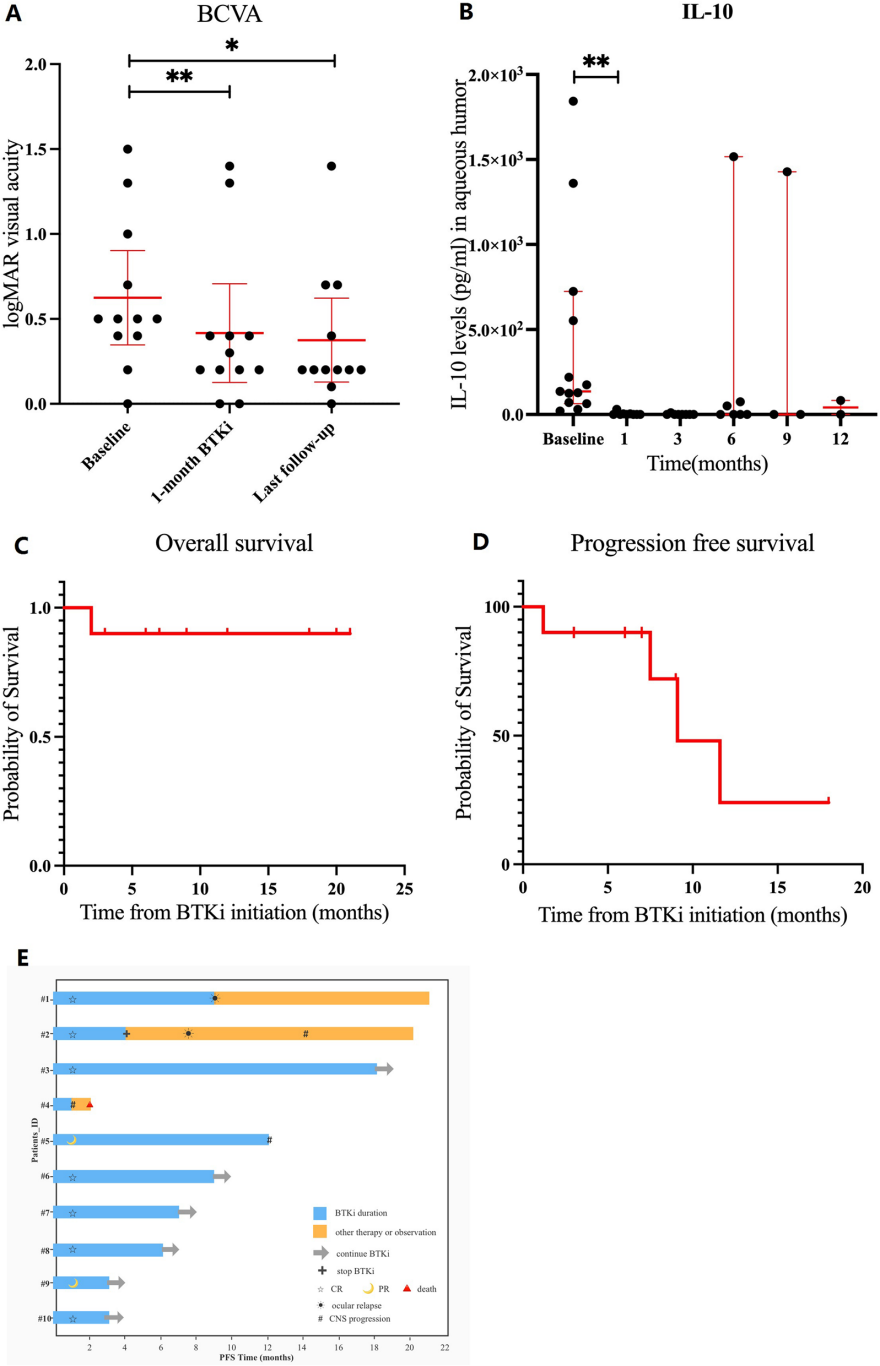
Overview of the efficacy of Bruton’s tyrosine kinase inhibitors (BTKi) in the treatment of 10 patients with vitreoretinal lymphoma. **A** Best-corrected visual acuity (BCVA) comparison between baseline and during follow-up with red lines indicating the mean and 95% confidence interval. **B** Interleukin (IL)-10 levels in aqueous humor at baseline and during follow-up with red lines showing the median and 95% confidence interval. **C** Overall survival (OS). **D** Progression-free survival (PFS). **E** Swimlane flowcharts illustrating the detailed efficacy information. Of the 10 patients, 7 achieved complete response (CR), 2 achieved partial response (PR), and one (#4) experienced central nervous system (CNS) progression after one-month BTKi treatment. Until the last follow-up, 2 of the 9 patients with disease control developed ocular relapse (#1 and #2), and 2 developed CNS progression (#2 and #5). The remaining 6 patients have durable response. *P < 0.05, **P < 0.01

Although VRL is a rare intraocular tumor, its incidence has been rising recently [2]. Based on previous encouraging results [10, 11], we hypothesized that BTKi may penetrate the blood-eye barrier for local tumor control and the blood–brain barrier for therapeutic or preventative activity in the CNS while minimizing systemic side effects. In this study, 90% patients achieved DC after 1-month BTKi therapy, and the median PFS was 8.3 months, with an estimated 60% patients without disease progression. In addition, the AH IL-10 level appeared to be a valuable marker in the follow-up of the disease. Several limitations existed concerning our study, including small sample size and relatively short follow-up periods.

In conclusion, targeting BTK in VRL is viable, and our findings could pave the way for a paradigm change in VRL therapy choices. A well-designed prospective study in a larger cohort of patients is needed to validate our findings.

## Supplementary Information

**Additional file 1: Table S1. **Characteristics of patients with PVRL and PCNSL with vitreoretinal involvement.

**Additional file 2: Table S2. **Diagnostic test results of vitreoretinal lymphoma patients.

**Additional file 3. **Additional methods.

## Data Availability

All data generated or analyzed during this study are included in this published article.

## Abbreviations

AH: Aqueous humor
BCVA: Best-corrected visual acuity
BTK: Bruton’s tyrosine kinase
BTKi: BTK inhibitor
DC: Disease control
IL: Interleukin
PCNSL: Primary central nervous system lymphoma
PFS: Progression-free survival
VRL: Vitreoretinal lymphoma
