# Supplementary material for: Targeting Bruton’s tyrosine kinase in vitreoretinal lymphoma: an open-label, prospective, single-center, phase 2 study
Source: Exp Hematol Oncol. 2022 Nov 8;11:95. doi: 10.1186/s40164-022-00354-2 (PMC9644621; doi:10.1186/s40164-022-00354-2)
Supplement: Supplementary file 1 — Additional file 1: Table S1. Characteristics of patients with PVRL and PCNSL with vitreoretinal involvement. [file 40164_2022_354_MOESM1_ESM.doc]

| **Patient** | **Age (years)** | **Sex** | **Primary-recurrent sites (if any)** | **Involved eye(s)** | | **Ocular presentation*** | **Previous treatment** | **Time to ocular relapse after previous treatment**  **(months)** | **BTKi^** |
| --- | --- | --- | --- | --- | --- | --- | --- | --- | --- |
| **#1** | 41 | F | PCNSL-O | RE | a | | MTX-based systemic chemotherapy  IVMTX (BE) | 20 | Z, O |
| **#2** | 49 | F | PCNSL-O | BE | a | | MTX-based systemic chemotherapy  IVMTX (BE) | 2 | Z |
| **#3** | 73 | F | PVRL | RE | a, b | | No | — | O |
| **#4** | 54 | F | PVRL-CNS\O | LE | a, b | | IVMTX (BE) | 4 | Z |
| **#5** | 47 | M | PVRL | BE | a, b | | No | — | Z, O |
| **#6** | 59 | M | PCNSL-O | BE | a | | MTX-based systemic chemotherapy  Temozolomide | 4 | O |
| **#7** | 74 | F | PVRL | RE | a | | No | — | O |
| **#8** | 30 | F | PCNSL-O | LE | a, b | | Temozolomide  Autologous stem cell transplantation | 9 | I |
| **#9** | 69 | M | PVRL | LE | a, b | | No | — | O |
| **#10** | 50 | F | PCNSL-O | LE | a, b | | MTX-based systemic chemotherapy  IVMTX (BE) | 1 | O |
| BTKi, Bruton tyrosine kinase inhibitors; BE, both eyes; F, female; IVMTX, intravitreal methotrexate; LE, left eye; M, male; O, ocular; PCNSL, primary central nervous system lymphoma; PVRL, primary viteroretinal lymphoma; RE, right eye. *a= vitreous opacity, b= retinal infiltration  ^BTKi: I=ibrutinib, Z=zanubrutinib, O=orelabrutinib. | | | | | | | | | |

Table S1. Characteristics of patients with PVRL and PCNSL with vitreoretinal involvement
