# Supplementary material for: Targeting Bruton’s tyrosine kinase in vitreoretinal lymphoma: an open-label, prospective, single-center, phase 2 study
Source: Exp Hematol Oncol. 2022 Nov 8;11:95. doi: 10.1186/s40164-022-00354-2 (PMC9644621; doi:10.1186/s40164-022-00354-2)
Supplement: Supplementary file 2 — Additional file 2: Table S2. Diagnostic test results of vitreoretinal lymphoma patients. [file 40164_2022_354_MOESM2_ESM.doc]

Table S2 Diagnostic test results of vitreoretinal lymphoma patients

| **Patient** | **CNS biopsy** | **Vitreous smear** | **IL-10/IL-6>1** | **B-cell clonality via FCM analysis** | **IgH gene rearrangement** |
| --- | --- | --- | --- | --- | --- |
| **#1** | **+** | NP | **+** | NP | NP |
| **#2** | **+** | NP | **+** | NP | NP |
| **#3** | NP | atypical cells | **+** | **+** | **-** |
| **#4** | + | atypical cells | **+** | **+** | **+** |
| **#5** | NP | **-** | **+** | **+** | **+** |
| **#6** | **+** | NP | **+** | NP | NP |
| **#7** | NP | atypical cells | **+** | **+** | **+** |
| **#8** | **+** | NP | **+** | NP | NP |
| **#9** | NP | **-** | **-** | **+** | **+** |
| **#10** | **+** | NP | **+** | NP | NP |
| CNS, central nervous system; FCM, flow cytometry; IgH, immunoglobulin heavy chain; IL, interleukin; NP, not performed. | | | | | |
