# Supplementary material for: Targeting Bruton’s tyrosine kinase in vitreoretinal lymphoma: an open-label, prospective, single-center, phase 2 study
Source: Exp Hematol Oncol. 2022 Nov 8;11:95. doi: 10.1186/s40164-022-00354-2 (PMC9644621; doi:10.1186/s40164-022-00354-2)
Supplement: Supplementary file 3 — Additional file 3. Additional methods. [file 40164_2022_354_MOESM3_ESM.docx]

**METHODS**

***Patients***

This study enrolled patients with DLBCL-VRL who presented to Beijing Tongren Hospital between October 2020 and April 2022. All individuals had a confirmed diagnosis of VRL based on vitreous and/or brain biopsy results. Patients identified by brain biopsy must also have a suspicious clinical picture (vitreoretinal abnormalities) and an interleukin 10 (IL-10)/IL-6 ratio of > 1.0 in intraocular fluid; otherwise, further vitreous biopsy is needed. To make the diagnosis, all vitreous biopsy samples were processed for cytologic examination, cell surface marker determination by flow cytometry, and monoclonality detection by gene rearrangement. Malignant cytology or at least two positive findings from four tests, including the detection of atypical cells by cytology, IL-10/IL-6 > 1, IgH gene rearrangement, or flow cytometry, serve as the basis for VRL diagnosis [1].

At the time of diagnosis, all patients underwent a systemic work-up that included a brain contrast-enhanced magnetic resonance imaging (MRI), a whole-body positron emission tomography computerized tomography (PET-CT) scan, and a bone marrow biopsy or lumbar puncture as needed. Patients were excluded from the trial if lymphoma was discovered outside of the eye and CNS. Patients with pretreatment (unexposed to BTK inhibitors) or concomitant CNS lymphoma were allowed to participate in this trial, but those with an expected survival time of less than three months were excluded.

***Study design and treatment***

This was an open-label, prospective, single-center, phase 2 study approved by the IRB of Beijing Tongren Hospital and conducted according to the Declaration of Helsinki and Good Clinical Practice (ChiCTR2000037921). All the patients or their guardians signed a written informed consent form.

All patients were treated with orally BTK inhibitors monotherapy (ibrutinib 560mg once daily, zanubrutinib 160mg twice daily or orelabrutinib 150mg daily), until disease progression or unacceptable toxicity.

***Assessment of therapeutic response and toxicity***

The therapeutic response assessment was scheduled to start after 1 month of treatment and was followed up every 3 months thereafter. At each visit, best-corrected visual acuity (BCVA), ophthalmoscope, fundus photography, optical coherence tomography (OCT), cytokine analysis (IL-10 and IL-6) in the aqueous humor (AH) using a Cytometric Bead Array (normal range: <5pg/ml), and brain MRI were assessed. The response was evaluated using the following criteria:

1. Complete response (CR), complete disappearance of lymphomatous infiltrates within the eye or CNS as determined by post-treatment ophthalmological examination, brain MRI scan, and normal IL-10 levels in the AH;
2. Partial response (PR), >50% reduction of lymphomatous infiltrates and IL-10 levels in the AH;
3. Stable disease (SD), no or <50% reduction of lymphomatous infiltrates and IL-10 levels in the AH;
4. Progressive disease (PD), deterioration of previous ocular findings or emerging lesions in the eye or CNS.

Toxicity was assessed according to the Common Terminology Criteria for Adverse Events (AE) version 4. Any AEs meeting seriousness criteria were reported.

***Outcomes***

The primary endpoint was the disease control (DC) rate, including CR, PR, and SD after 1 month of treatment. The secondary endpoints were toxicity, overall survival (OS), and progression-free survival (PFS). The OS was computed from the date of BTK inhibitor initiation to the date of the last follow-up or death. PFS was calculated as the period from the onset of BTK inhibitors to lymphoma relapse, death, or the final follow-up. Relapse was defined as the reappearance of lymphoma cells in any location (vitreous cavity, retina, or CNS) in individuals who had previously proven remission.

***Statistical analysis***

Quantitative data are presented as mean (± standard deviation [SD]) or median (interquartile range [IQR]), as appropriate, and qualitative data as absolute numbers and percentages. Comparison of BCVA before and after treatment was performed using a paired-samples T test, and BCVA was converted to the logarithm of the minimum angle of resolution (Log MAR). Comparison of IL-10 levels before and after treatment was performed using a Wilcoxon matched-pairs signed rank test. OS and PFS were estimated by the Kaplan-Meier method. Statistical analysis was performed using GraphPad Prism V.9 (California, USA). P value <0.05 was considered statistically significant.

**DISCUSSION**

The goals of VRL treatment are to achieve ocular tumor control as well as to minimize tumor-specific death owing to subsequent CNS disease. Intravitreal methotrexate (IVMTX) is presently the most popular local treatment method for VRL because of its low incidence of adverse events and high remission rate. In a large series, Habot-Wilner et al. [2] reported the effects of IVMTX in 134 eyes of 81 patients who received a mean of 19 injections within a year. All patients achieved CR with five injections, and two patients had ocular recurrence. Although IVMTX can control intraocular lesions, it is insufficient to prevent CNS involvement, with 56% of VRL patients developing CNSL in their study.

Is there a possibility to improve CNS development by systemic chemotherapy? A 17-Center European Collaborative Study [3] on 78 patients reported the outcomes of local and systemic chemotherapy used for PVRL in the prevention of subsequent CNSL. They indicated that systemic chemotherapy had not been demonstrated to prevent CNSL than local treatment (43% vs. 39%) and was associated with more severe adverse effects.

References:

[1] Tanaka R, Kaburaki T, Taoka K, et al. More Accurate Diagnosis of Vitreoretinal Lymphoma Using a Combination of Diagnostic Test Results: A Prospective Observational Study. *Ocular immunology and inflammation*. Apr 1 2021:1-7. doi:10.1080/09273948.2021.1873394

[2] Habot‐Wilner Z, Frenkel S, Pe’er J. Efficacy and safety of intravitreal methotrexate for vitreo‐retinal lymphoma – 20 years of experience. Br J Haematol. 2021;194(1):92–100.

[3] Riemens A, Bromberg J, Touitou V, Sobolewska B, Missotten T, Baarsma S, et al. Treatment Strategies in Primary Vitreoretinal Lymphoma: A 17-Center European Collaborative Study. JAMA Ophthalmol. 2015;133(2):191.
